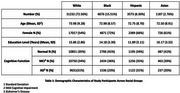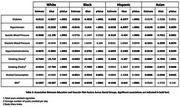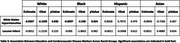# Education is associated with vascular risk factors and cerebrovascular disease burden in racially diverse individuals on the Alzheimer's disease spectrum

**DOI:** 10.1002/alz70860_104875

**Published:** 2025-12-23

**Authors:** Shima Raeesi, Cassandra Morrison, Mahsa Dadar

**Affiliations:** ^1^ McGill University, Montreal, QC, Canada; ^2^ Carleton University, Ottawa, ON, Canada; ^3^ Douglas Mental Health University Institute, Montréal, QC, Canada; ^4^ Department of Psychiatry, McGill University, Montréal, QC, Canada

## Abstract

**Background:**

Education has been shown to mitigate the risk of Alzheimer's dementia (AD). While the link between education and cognitive reserve has been investigated, another potential pathway through which education can impact cognitive decline could be through its influence on vascular risk factors which in turn lead to cerebrovascular pathology such as white matter hyperintensities (WMHs) and infarcts. The prevalence of these factors varies across racial groups, highlighting the importance of understanding disparities in education and health outcomes in these populations.

**Method:**

This study analyzed data from the National Alzheimer's Coordinating Center (NACC), including 42,668 participants aged 55 and older from diverse racial backgrounds: White (*n* = 31,232), Black (*n* = 6,676), Asian (*n* = 3,573), and Hispanic (*n* = 1,187) (Table 1). Linear mixed‐effects models were employed to investigate the relationship between education and vascular risk factors (i.e., diabetes, hypertension, hypercholesterolemia, systolic and diastolic blood pressure (BP), smoking, alcohol consumption, and body mass index; BMI), WMHs, and lacunar infarcts. Age, sex, and diagnostic status (cognitively normal, mild cognitive impairment, and AD) were added as covariates in the models.

**Result:**

Higher education was significantly associated with lower rates of diabetes, hypertension, hypercholesterolemia, and smoking in most racial groups (*p* < .0001). In White and Hispanic groups, strong negative associations were seen for all risk factors (*p* < .0001), except diastolic BP and alcohol consumption. Among Black individuals, significant relationships were observed for all risk factors (*p* < .0001), except for systolic and diastolic BP. In Asian individuals, education was associated with all vascular risk factors (*p* < .05) except BMI and diabetes (Table 2). Furthermore, higher education was significantly associated with a lower WMH burden in White (*p* = 0.03) and Black (*p* = 0.02) individuals (Table 3). No significant relationship was observed between education and lacunar infarcts in any race.

**Conclusion:**

While higher education was overall negatively associated with vascular risk factors in all racial groups, certain risk factors showed different associations with education across different groups. Racial disparities were also found in the relationship between education and cerebrovascular disease markers, suggesting that while education is generally associated with better health outcomes, its impact varies across racial groups.